# Case report: A novel surgical technique for rapid valve-in-ring implantation into the native aortic annulus during left ventricular assist device implantation

**DOI:** 10.3389/fcvm.2023.1091420

**Published:** 2023-04-06

**Authors:** Yuriy Pya, Abdurashid Mussayev, Svetlana Novikova, Makhabbat Bekbossynova, Serik Alimbayev, Nail Khissamutdinov, Timur Kapyshev, Aidyn Kuanyshbek, Timur Lesbekov

**Affiliations:** ^1^ Department of Cardiac Surgery, National Research Cardiac Surgery Center, Astana, Kazakhstan; ^2^ Department of Interventional Cardiology, National Research Cardiac Surgery Center, Astana, Kazakhstan; ^3^ Department of Cardiology, National Research Cardiac Surgery Center, Astana, Kazakhstan; ^4^ Department of Anesthesiology, Resuscitation and Intensive Care (Adult), Astana, Kazakhstan

**Keywords:** left ventricular assist device (LVAD), native aortic valve, ring annuloplasty, transcatheter aortic valve replacement, valve-in-ring, balloon expandable valve, heart failure

## Abstract

The implantation of a left ventricular assist device (LVAD) has become an essential requirement for managing patients with end-stage heart failure. However, aortic valve insufficiency is a contraindication for LVAD implantation in patients with end-stage heart failure, partly because of the decreasing efficiency of mechanical circulatory support and the eventual development of right ventricular failure. Herein, we present the first case of performing transcatheter aortic valve replacement in valve-in-ring along with LVAD implantation for the treatment of a 60-year-old male suffering from refractory heart failure due to dilated cardiomyopathy and pure aortic insufficiency in need of a new aortic bioprosthesis. A balloon-expandable bioprosthetic transcatheter heart valve was implanted into a previously sewn annulus ring into the aortic root *via* transaortic access. Subsequently, a centrifugal-flow LVAD was implanted. Postoperatively, the patient was in New York Heart Association Functional Class (NYHA) II with 6-min walk test of 310 m. The patient has completed 6 months of follow-up with no events. This novel and feasible surgical technique reduced the cardiopulmonary bypass time and duration of surgery. Furthermore, it avoids the risk of redo sternotomy and decreases the chances of paravalvular leakage and worsening of aortic regurgitation.

## 1. Introduction

In patients with end-stage heart failure who are refractory to medical treatment and non-definitive surgical management, left ventricular assist device (LVAD) is an effective alternative to heart transplantation, especially with limited donor availability (1, 2).

Recent studies show that continuous flow LVADs can improve the survival of end-stage heart failure patients and have been in current clinical use as a destination therapy or as a bridge-to-transplant (1–3). However, the use of LVAD has been reported to cause aortic insufficiency (AI), as early as 6 months, requiring continuous hemodynamic monitoring of the aortic valve (AV) post implantation (1, 3). The pathophysiology of this LVAD-associated AI is partly related to the impact of the LVAD, causing degenerative changes and remodeling of the AV (4, 5). In addition, the following AV hemodynamic abnormalities have been implicated in LVAD-associated AI: (i) reduced AV opening, (ii) local flow stasis, (iii) high shear stress, (iv) inversion of the transvalvular pressure gradient, and (v) pancyclic pressure gradient (4). LVAD implantation also induces anatomical alterations, such as retraction and aortic cusp malcoaptation, fibrosis, and aortic wall remodeling, which ultimately lead to AI (5). Surgical repair is the only option in such cases, and AV closure (AVC) or replacement (AVR) are considered safe and reliable for treating conditions that could lead to right ventricular failure (5–7).

The surgical repair of the AV in the context of LVAD-associated anatomical remodeling generally includes ring annuloplasty (8). However, reports show that AV regurgitation can develop even after AV repair within a few months of LVAD implantation with concomitant annuloplasty (3, 8, 9). Hence, concomitant AVR with ring annuloplasty during LVAD implantation has been adopted and reported with feasible outcomes (8, 9). Minimizing or mitigating the risks associated with the surgical settings, in addition to increased cardiopulmonary bypass time, is highly desirable. In lieu of such challenges, transaortic transcatheter aortic valve replacement (TAVR) has been considered a suitable treatment, which aids in preventing ischemia at the cross-clamping site. The increased evidence of positive clinical outcomes of TAVR with improved transcatheter heart valve (THV) designs, especially for highly comorbid patients with a high-surgical-risk status, has encouraged more clinicians to consider its use for the management of severe aortic regurgitation (AR) patients, including those with AI (10).

We present a novel technique that combines the implantation of a THV with a newly attached annuloplasty ring, valve-in-ring (ViR) followed by concomitant LVAD implantation. In this method, THV deployment is performed through the transapical route before LVAD implantation; we used a balloon-expandable biological transcatheter valve prosthesis, Myval THV (Meril Life Sciences, Pvt. Ltd., India). This Conformité Européenne (CE)-marked THV is composed of a cobalt alloy (MP35N) frame with a hybrid honeycomb cell design. This case report presents the novel surgical technique that modifies and improves upon the previous techniques of AV repair.

## 2. Case report

**Figure 1:**
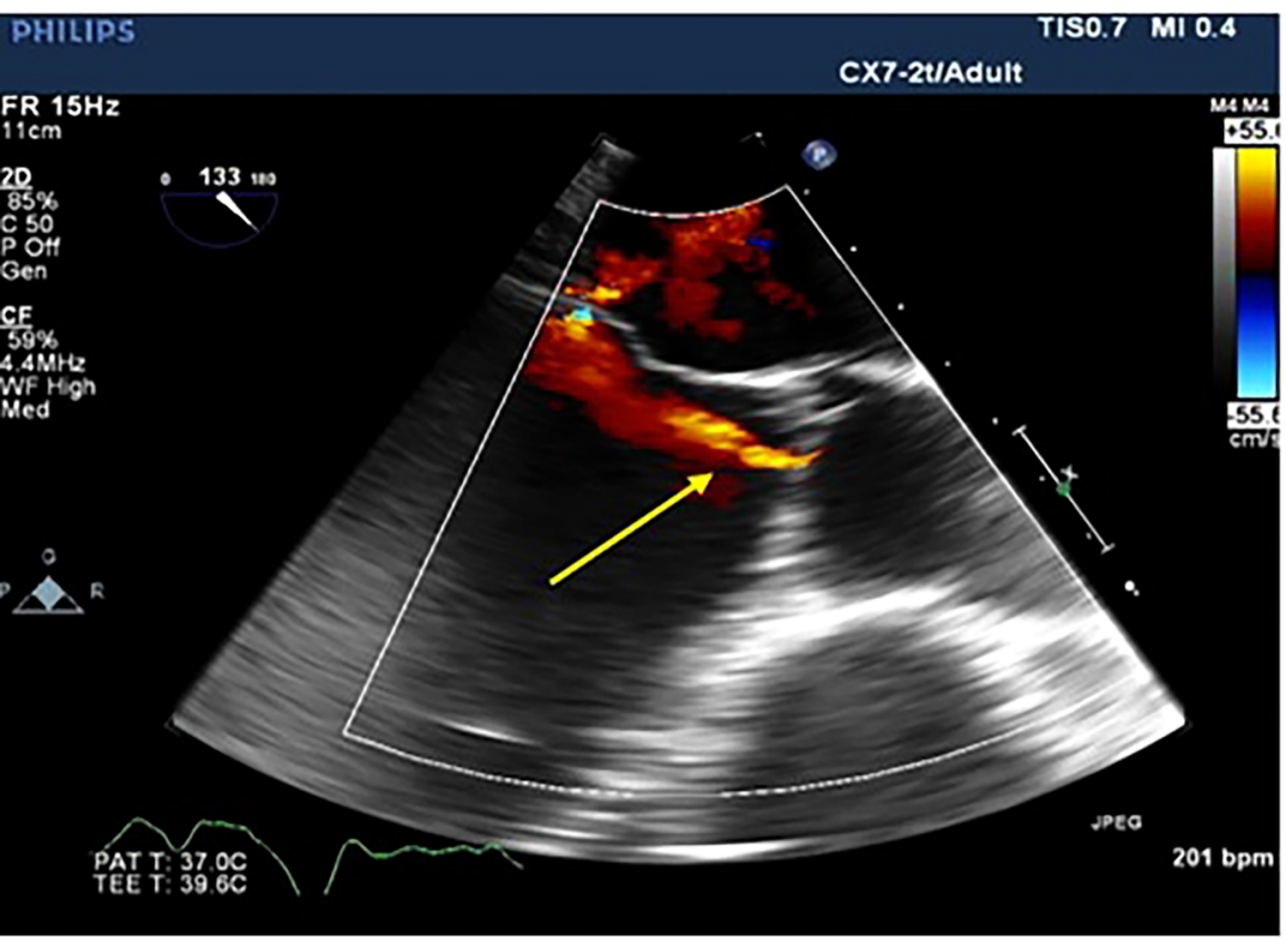
Transesophageal echocardiographic long access view of the aortic valve showing aortic regurgitation jet (yellow arrow).

A 60-year-old male with refractory heart failure due to idiopathic dilated cardiomyopathy [New York Heart Association Functional Class (NYHA) III., INTERMACS IV] was admitted to our institution. The patient had a history of heart failure since 2012 that was treated by implanting a cardiac resynchronization therapy defibrillator (CRT-D) in 2012 and pulse generator replacement in 2017. Echocardiography demonstrated a very low left ventricular ejection fraction (LVEF) of 23%, dilated left ventricle with end-diastolic volume of 424 ml and an elevated left ventricular end-diastolic diameter (LVEDD) of 8.7 cm, severe mitral regurgitation (MR), and mild to moderate eccentric AI (Figure 1). The etiology of dilated cardiomyopathy was confirmed as idiopathic after ruling out the common causes. In view of nonavailability of donors and long waiting period for transplant, LVAD was considered in this patient as a bridge-to-transplant. The patient's clinical profile suggested his candidature for LVAD implant with concomitant AVR; hence, the patient was recommended for the same. Preoperative multislice computed tomography (MSCT) analysis was performed to assess the feasibility for a transfemoral procedure using the Structural Heart v.10.3 software (NRCSC). MSCT analysis revealed, the aortic annulus area was 702.1 mm^2^, area-derived annular diameter was 29.9 mm, annular perimeter was 96.5 mm, and the right and left coronary artery heights were 15.4 and 13.3 mm, respectively (Supplementary Figures S1 and S2). The aortic root analysis was validated using the 3mensio software (Pie Medicals, Netherlands) at an independent Core lab (TAVI Core Lab, Meril Life Sciences Pvt. Ltd., India).

### 2.1. Surgical procedure

**Figure 2:**
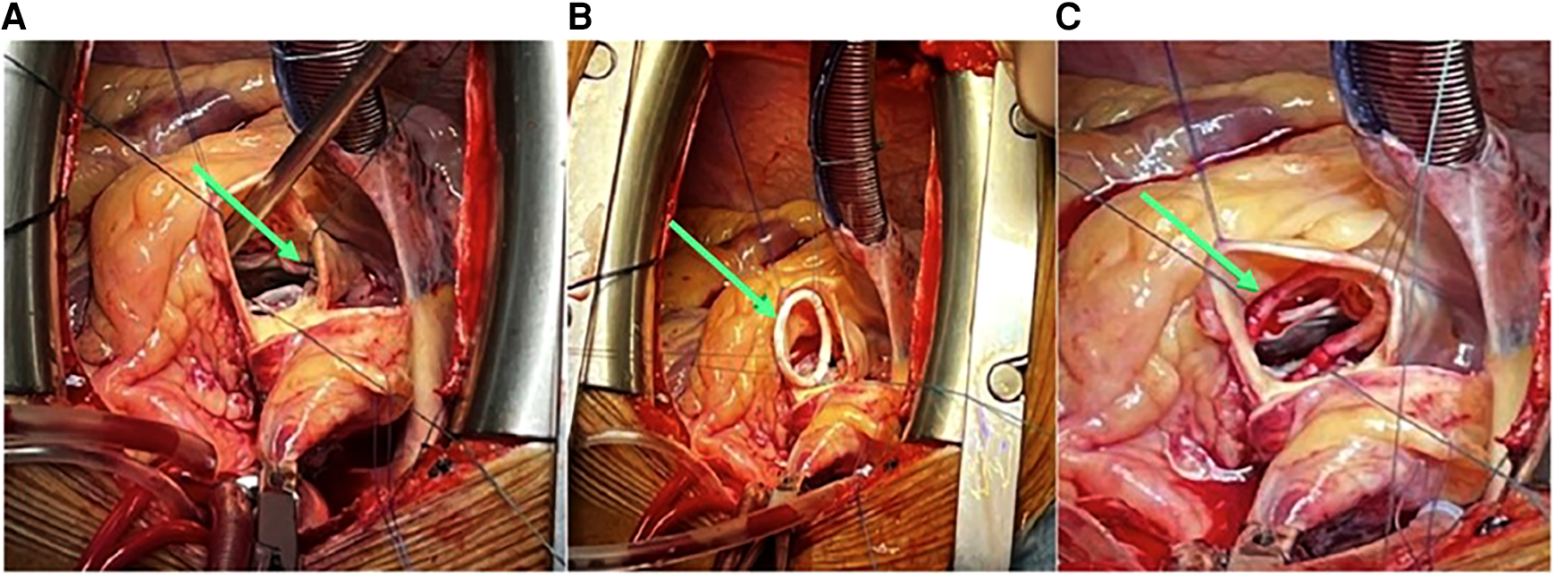
(**A**) Native annulus stitching with 2/0 Ethibond sutures (green arrow). (**B**) Carbomedics Annuloflex flexible ring #32 stitching (green arrow). (**C**) Carbomedics Annuloflex ring #32 positioning and fixation (green arrow).

The surgery was carried out using median sternotomy under cardiopulmonary bypass support with aortic cross-clamping after inducing cardioplegia. The pericardium was cut open and by oblique aortotomy, the aortic leaflets were excised and the valve was exposed. The basal attachment of AV leaflets was sewn using an Ethibond 2/0 suture (Johnson & Johnson Medical N.V., Belgium) to the ventricular myocardium (Valvar Hinge) using three stitches, each leaflet bearing one stitch. A flexible annuloplasty ring [CARBOMEDICS AnnuloFlex TM32 flexible ring (Sorin Group Italia S.r.L., Italy)] was fixed with the native annulus using the same stitches to provide a sturdy base for valve replacement, as illustrated in Supplementary Figure S3 (11) and in Figure 2.

**Figure 3:**
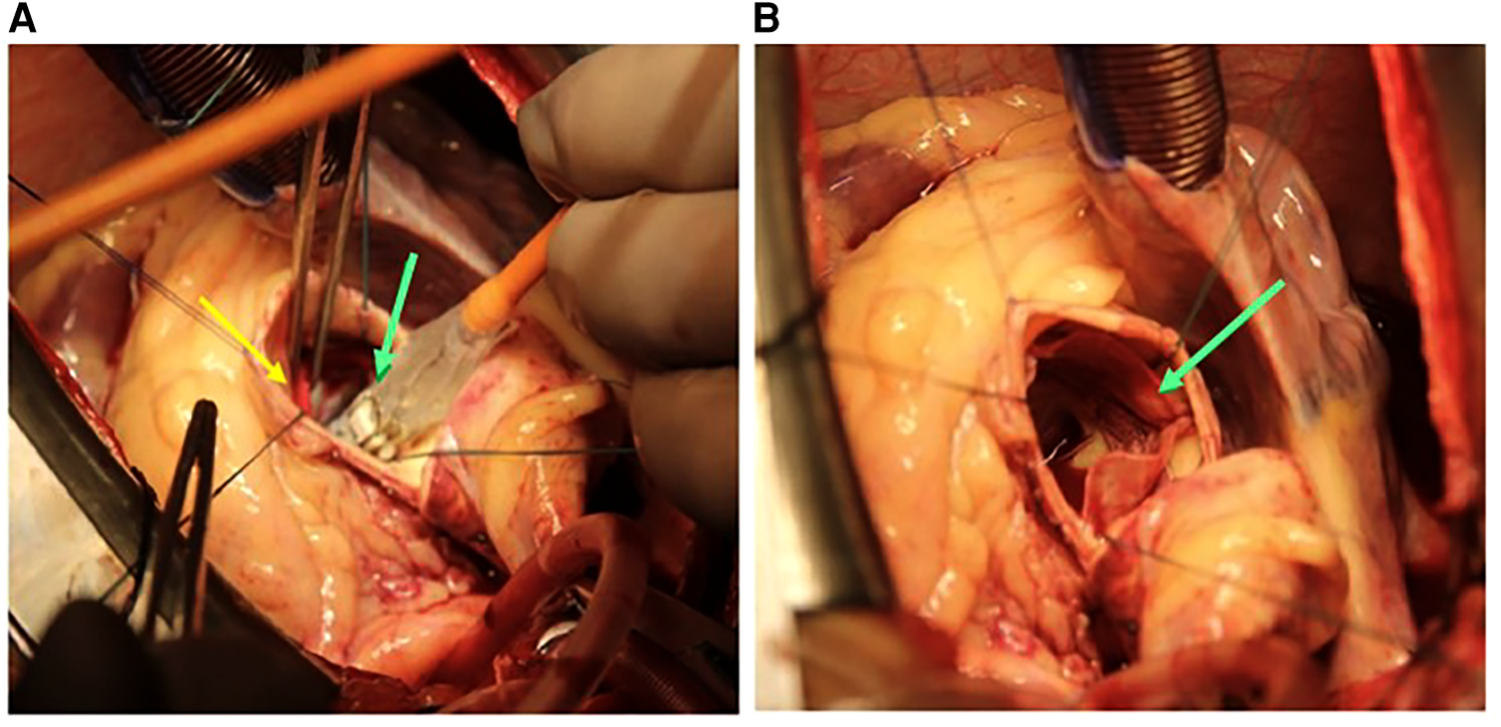
(**A**) Deployment of the 30.5 mm Myval THV (green arrow) positioning into the ring (yellow arrow). (**B**) Deployed THV (green arrow). THV, transcatheter heart valve.

Thereafter, the extra-large size (30.5 mm) of the balloon-expandable THV was deployed such that it provides a coverage of 70% aortic root and 30% left ventricular outflow tract (LVOT). The balloon-expandable THV was dilated using the Navigator THV Delivery System filled with a nominal volume of 36 ml saline, as shown in Figure 3. The deployment was performed according to the manufacturer's instructions. Subsequently, the aortic cross-clamp was removed and the Navigator THV Delivery System was retracted. The total cross-clamping time was 15 min.

**Figure 4:**
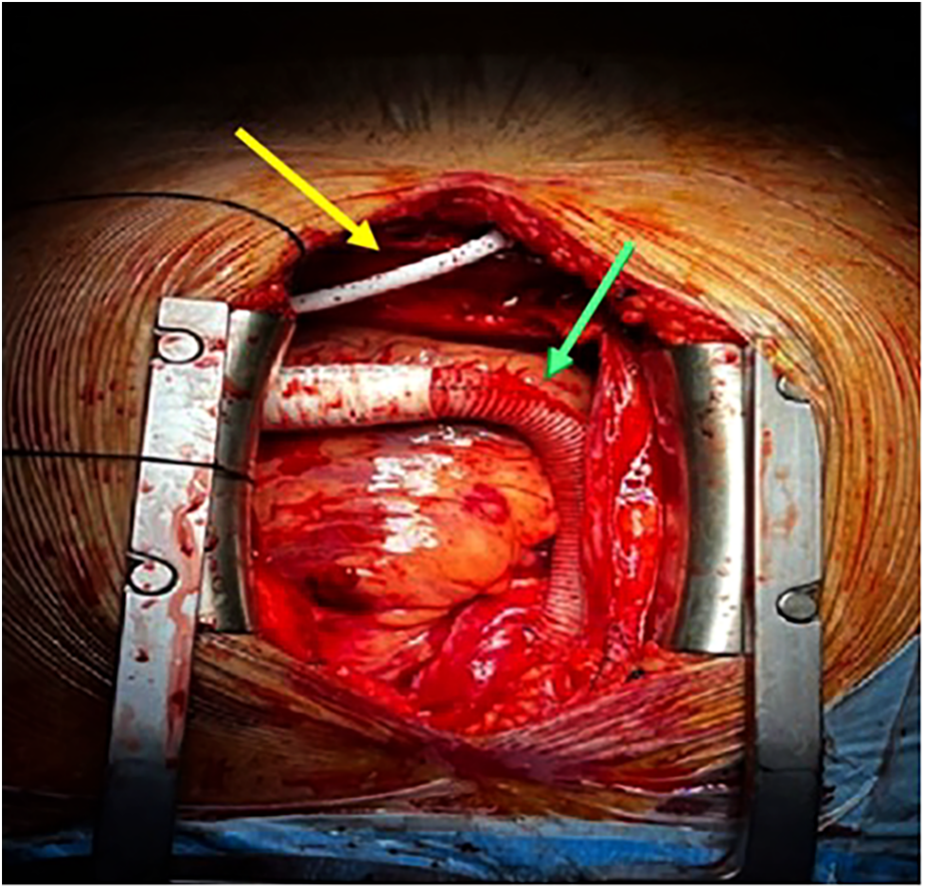
Implantation of HeartMate 3™ LVAD, LVAD HM3 outflow graft (green arrow), and LVAD HM3 driveline cable (yellow arrow). LVAD, left ventricular assist device.

After a successful TAVR, the HeartMate 3™ LVAD (Abbott Vascular, United States) was implanted (Figure 4) through the standard apical to ascending aorta approach.

### 2.2. In-hospital outcomes

The patient was removed from cardiopulmonary bypass after 100 min and operative time (measured as skin-to-skin time) was 190 min. As a result, the patient was extubated 4 h later and stayed in the ICU for 1 day. The patient was discharged and taken to the rehabilitation department 3 days after the surgery. Postoperative echocardiography and fluoroscopy results confirmed the adequate valve positioning without any transvalvular or periprosthetic regurgitation, as shown in Supplementary Figure S4. Postoperatively, the patient was in NYHA II with a 6-min walk test of 310 m. Currently, the patient has completed 6 months of follow-up with no events.

## 3. Discussion

In this case, a concomitant annuloplasty and implantation of a transcatheter AV were performed along with LVAD placement in a patient having stage D heart failure with reduced ejection fraction (23%). Despite the hybrid procedure, we achieved satisfactory procedural outcomes with no device-related or procedure-related complications including deployment failure or postoperative residual AR. In this case, we performed the first ever direct transaortic TAVR using balloon-expandable THV into a newly sewn flexible annuloplasty ring (ViR) on the native AV annulus during the LVAD implantation procedure. The considerable decrease in ischemia time is the key benefit of this new procedure. This technique resolves the issues of late severe AR associated with LVAD implantation, including the risks of redo sternotomy after LVAD, and retains the structural suitability for a possible need of future TAVR. In addition, the availability of extra-large sizes (30.5 and 32 mm) of this balloon-expandable THV enabled better size selection, given that if these sizes were not available, the 29 mm THV would have been used although being excessively oversized, which might not have generated optimal outcomes.

The selection of the treatment modality is a complex decision, as there is a lack of consensus regarding the optimal management of LVAD-related AR. Chung et al. performed ViR implantation in a patient with severe LVAD-associated AR who received a self-expanding AV bioprosthesis and reported excellent post-procedural hemodynamic outcomes. This case report offers encouraging evidence for considering the suitability of TAVR at a later stage following LVAD implantation in such critical heart failure patients (9). Dranishnikov et al. performed simultaneous AVR and LVAD implantation in 19 patients, in whom they reported longer cardiopulmonary bypass times for AVR cases using biological AV with prior LVAD, compared to patients with LVAD alone (12). They also stated that simultaneous AVR with LVAD implantation does not worsen the prognosis (12), which provides considerable impetus to performing concomitant AVR with LVAD implantation for treating stage D heart failure patients with AI (12). The use of a bioprosthetic AV in cases of LVAD-associated AI has been reported with acceptable perioperative and early (30-day) outcomes (9, 12, 13). Hence, a bioprosthetic, balloon-expandable THV was used for the ViR technique as a potential solution in our case.

Rapid deployment (RD)-AVR and TAVR have a shorter cross-clamping time and an overall procedural time, which minimizes the risk of intraoperative myocardial ischemia (5, 14, 15). Rahmanian et al. examined 163 patients undergoing RD-AVR and reported the mean cross-clamping and cardiopulmonary times as 55 ± 23 and 88 ± 38 min respectively, which were relatively lesser than those of the control group (77 ± 22 and 105 ± 38 min), respectively, whereas in-hospital mortality rates of both the RD-AVR and control groups were similar [1.8%, (*n* = 3/163); *p* = 1.000] (16). Similar outcomes were reported by White et al., who retrospectively observed patients undergoing RD-AVR and conventional AVR (295 patients in each group). Their key findings were as follows: (i) reduced cross-clamping (73.8 ± 37.5 and 107 ± 14.9 min) and procedural times (3.4 ± 1.0 and 3.7 ± 0.5 h); (ii) higher rates of new permanent pacemaker implantation leading to longer hospital stay in the RD-AVR group (∼7%); and (iii) similar rates of early myocardial infarction, stroke, and early mortality in both groups (17). Holloway et al. performed RD-AVR using a 23-mm Edwards INTUITY valve (Edwards Lifesciences, Irvine, CA, United States) in an AI patient undergoing concomitant LVAD implantation (14). In the case-series reported by Gangahanamaiah and Marasco (18), RD-AVR was performed using the Perceval valve (LivaNova, London, United Kingdom).

Overall, though RD-AVR is considered a safe treatment for end-stage heart failure, it is more expensive. Moreover, performing RD-AVR using balloon-expandable valves has not shown feasibility yet, since the newly inserted AV prosthesis exerts excessive radial force on the LVOT, which leads to the need for permanent pacemaker (18, 19). Moreover, a higher mortality rate has been reported for AV closure + repair with LVAD, compared to AVR with LVAD (6). AV repair in LVAD-assisted surgery fails if TAVR is required in future. To overcome these case-specific challenges, an initial ring annuloplasty to repair the AV with LVAD was performed by Chung et al. which allows the prospects of a subsequent ViR TAVR procedure (9).

The use of a balloon-expandable biological THV in the cases of LVAD failure-associated AI has rarely been reported, particularly for aortic ViR procedures. We believe that more data should be accumulated from high-volume cardiology centers by surgeons who may consider using this technique to perform aortic ViR procedures in LVAD recipients having AR and/or AI to understand the clinical effectiveness of this technique.

### 3.1. Limitations

There are a few limitations of this technique. The first is the absence of a specific aortic annulus ring constructed for this procedure, which might affect the stabilization of the aortic annulus or may not achieve the leaflet coaptation. The second is that our novel technique may not be suitable for treating congenital malformations of the aortic root, such as bicuspid aortic valve or Marfan syndrome.

### 3.2. Future directions

Patients with end-stage heart failure have limited treatment options and experience aortic valve dysfunction/insufficiency post-LVAD implantation. Furthermore, paucity of the literature on balloon-expandable valves provides an opportunity to investigate the field and afford patient-specific treatment options, if necessary. This cutting-edge technique offers a breakthrough for high-risk, younger patients and may serve as a potential platform for future interventions. In addition to dealing with aortic insufficiency, balloon-expandable valves also lower the need for a permanent pacemaker and improve hemodynamic outcomes, as seen in this case. This novel and feasible surgical technique shortens the ischemia time, avoids the risk of redo sternotomy, reduces the possibility of paravalvular leakage, and prevents the progression of aortic regurgitation.

## 4. Conclusion

This novel technique of direct transaortic transcatheter valve-in-ring with LVAD implantation can be considered in elderly, high-risk heart failure patients, particularly those with prior cardiac surgeries, complex valvular anatomy, and poor ejection fraction. Planning and execution of preoperative and perioperative strategies must be combined with vigilant postoperative management of an LVAD patient. The key benefit of this new procedure is that it enables the surgeons to reduce the ischemia time. Furthermore, the issues of late severe AR associated with LVAD implantation and redo sternotomy are resolved. The valve anatomy is maintained, thus allowing the possible scope for a future TAVR after LVAD if necessary, and decreasing the risk associated with multiple cardiothoracic surgeries and improving patient prognosis.

## Data Availability

The original contributions presented in the study are included in the article/**Supplementary Material**, further inquiries can be directed to the corresponding authors.

## Supplementary material

SUPPLEMENTARY FIGURE 1

Annulus (A) and Left (B) ventricular outflow tract measurement by multidetector row computed tomography. The resulting annular plane is seen.

SUPPLEMENTARY FIGURE 2

The heights of right (A) and left (B) coronary artery from basal plane measurement by multidetector row computed tomography.

SUPPLEMENTARY FIGURE 3

Pictorial representation of Ethibond sutures with flexible ring[11].

SUPPLEMENTARY FIGURE 4

A- Transcatheter heart valve leaflets (yellow arrow); Color Doppler with no signs of PVL; B- Annular ring (yellow arrow); Transcatheter heart valve (green arrow); Left ventricular assist device (blue arrow).
