# Supplementary figures and images for: Case report: A novel surgical technique for rapid valve-in-ring implantation into the native aortic annulus during left ventricular assist device implantation
Source: Front Cardiovasc Med. 2023 Apr 6;10:1091420. doi: 10.3389/fcvm.2023.1091420 (PMC10117784; doi:10.3389/fcvm.2023.1091420)

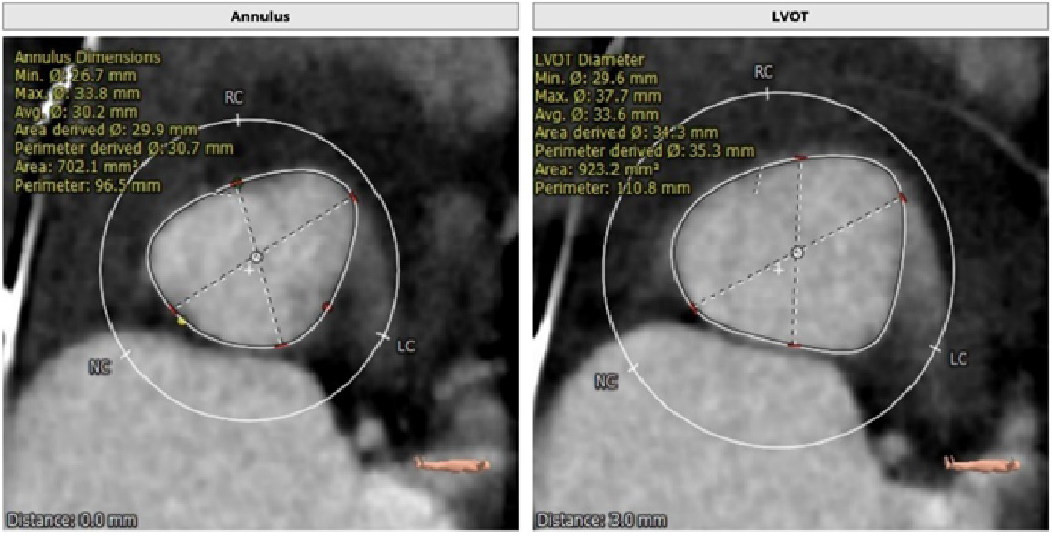

Supplement: Supplementary file 1 [file Image1.jpeg]

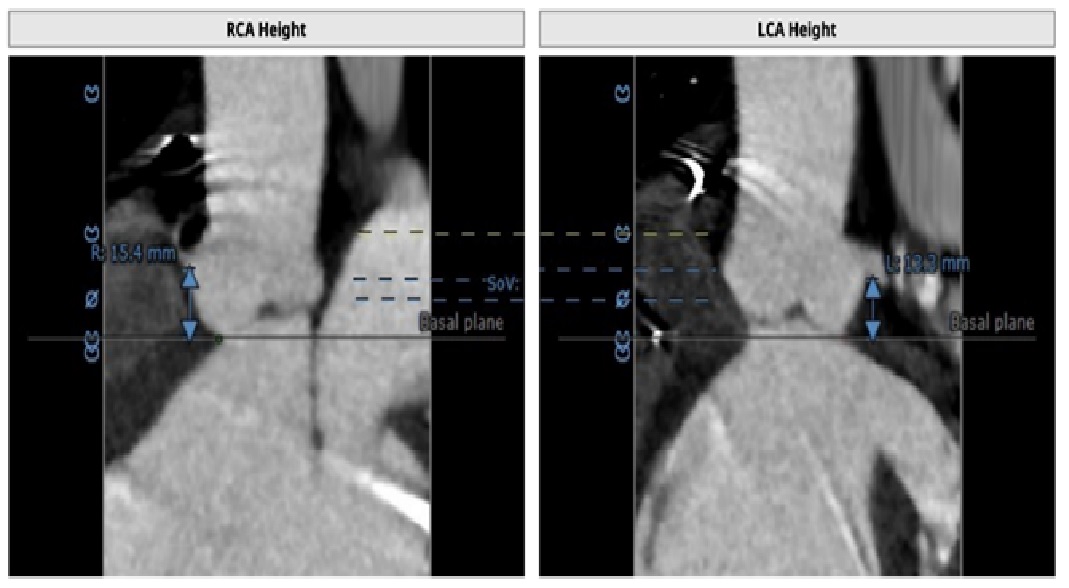

Supplement: Supplementary file 2 [file Image2.jpeg]

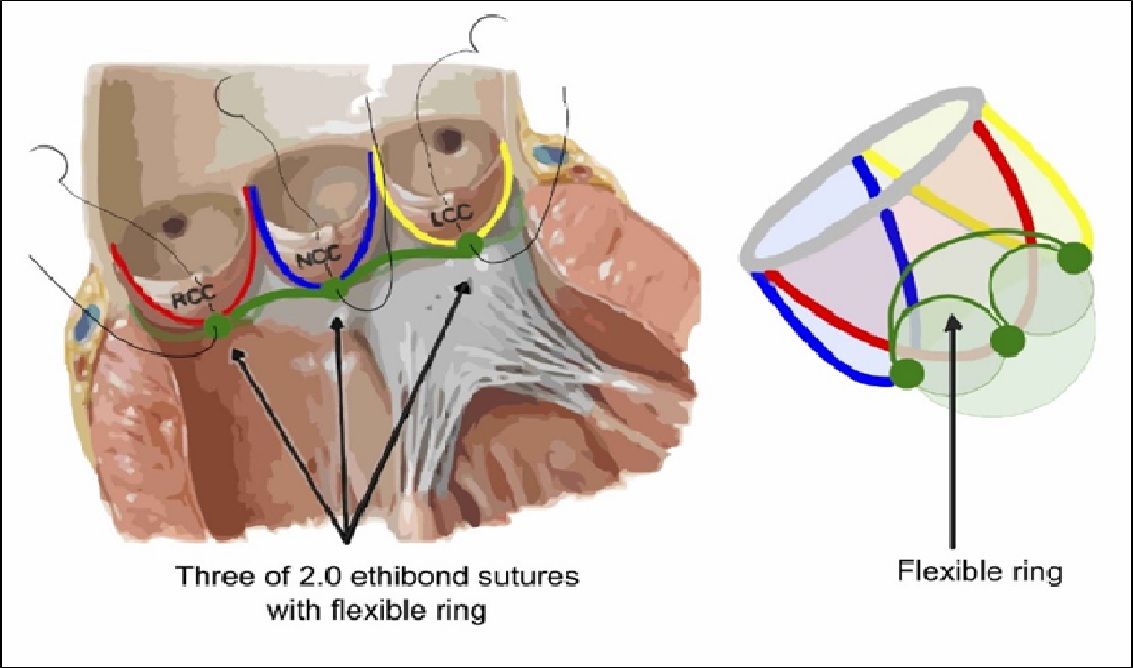

Supplement: Supplementary file 3 [file Image3.jpeg]

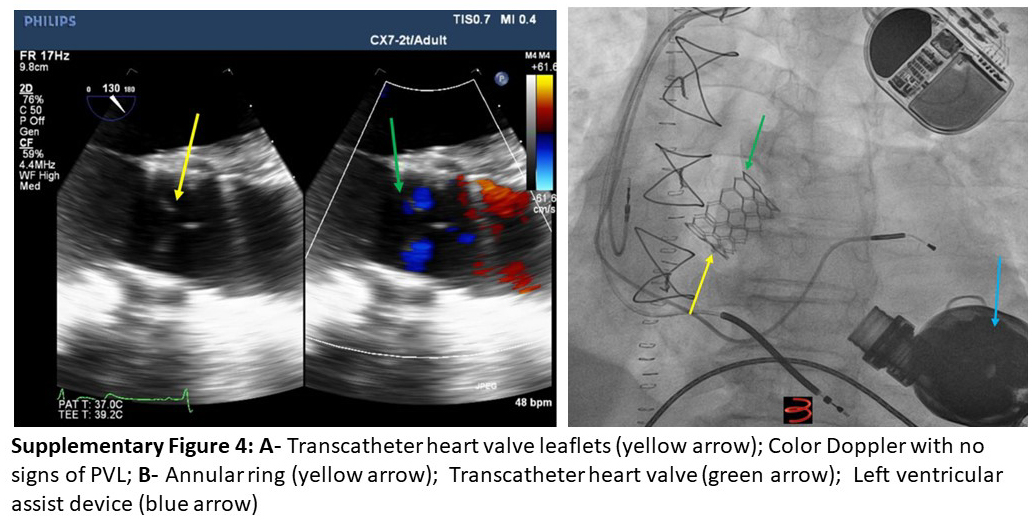

Supplement: Supplementary file 4 [file Image4.jpeg]
